# Raphin‐1 mediates the survival and sensitivity to radiation of pediatric‐type diffuse high‐grade glioma via phosphorylated eukaryotic initiation factor 2α‐dependent and ‐independent processes

**DOI:** 10.1002/1878-0261.70081

**Published:** 2025-07-09

**Authors:** Karin Eytan, Moshe Leitner, Amos Toren, Shoshana Paglin, Michal Yalon

**Affiliations:** ^1^ Pediatric Hemato‐Oncology Edmond and Lilly Safra Children's Hospital and Cancer Research Center, Sheba Medical Center Ramat Gan Israel; ^2^ Sackler School of Medicine Tel Aviv University Israel; ^3^ The Talpiot Medical Leadership Program Chaim Sheba Medical Center Ramat Gan Israel

**Keywords:** eIF2α phosphorylation, GRP78/BiP, pediatric‐type diffuse high‐grade glioma, radiation, raphin‐1, salubrinal

## Abstract

The primary treatment for fatal pediatric‐type diffuse high‐grade glioma (PED‐DHGG) which harbor the H3K27M or H3G34R/V mutation is radiation, but it provides only short‐term relief. Inhibitors of phosphorylated eIF2α (PeIF2α) phosphatase—namely raphin‐1 and salubrinal—decrease survival of PED‐DHGG cell lines and sensitize them to radiation. However, although both drugs increase PeIF2α, they have different effects on common targets and different targets altogether. Here, we aimed to identify PeIF2α‐phosphatase‐dependent and PeIF2α‐phosphatase‐independent molecular targets. Raphin‐1 but not salubrinal, decreased the level of BiP and CReP and increased that of DR5, in an ISRIB‐independent manner. Raphin‐1 induced similar changes in MEF^S51A^ cells and in irradiated PED‐DHGG, suggesting a PeIF2α‐independent contribution to raphin‐1's radiosensitizing effect. Importantly, while the expression of [S51D] eIF2α decreased the survival of PED‐DHGG and both raphin‐1 and salubrinal decreased the survival of MEF^WT^ cells, only raphin‐1 decreased the survival of mutant MEF^S51A^ cells. Our results suggest that the sensitivity of PED‐DHGG to raphin‐1 is mediated by both PeIF2α‐dependent and PeIF2α‐independent processes. Elucidating these processes could reveal targets for the development of drugs to overcome radiotherapy resistance of PED‐DHGG.

## Graphical abstract

Raphin‐1 reduces the survival of PED‐DHGG cells and enhances their radiation sensitivity through both PeIF2α‐dependent and PeIF2α‐independent mechanisms. Raphin‐1 sustains elevated levels of PeIF2α, contributing to its PeIF2α‐dependent effects. Additionally, raphin‐1 interacts with CReP to mediate a separate radiosensitizing pathway that operates independently of PeIF2α. This mechanism involves: Downregulation of BiP, upregulation of DR5 and activation of caspase‐8.

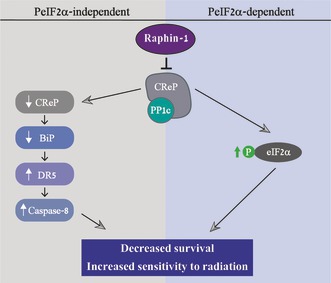

**Abbreviations**
BiP: binding immunoglobulin protein
ClpX: caseinolytic mitochondria matrix peptidase chaperone subunit X
CReP: constitutive repressor of eIF2α phosphorylation
DIPG: diffuse intrinsic pontine glioma
DR5: death receptor 5
GADD34: growth arrest and DNA damage‐inducible protein
ISRIB: integrated stress response inhibitor
MEF: mouse embryonic fibroblasts
PED‐DHGG: pediatric‐type diffuse high‐grade glioma
PeIF2α: phosphorylated eukaryotic initiation factor α
XBP1s: spliced x‐box binding protein‐1

## 1 Introduction

The H3K27M and H3G34R/V heterozygous somatic missense mutations characterize deadly pediatric‐type diffuse high‐grade glioma (PED‐DHGG). The H3K27M mutation is found in 80% of patients with diffuse midline glioma located in the pons (DIPG), cerebellum, thalamus, and spine [1, 2]. Due to their location, these tumors are inaccessible for surgical resection. Thus, despite ongoing development and testing of novel immunotherapy treatments (e.g., chimeric antigen receptor‐T cells [3, 4]) and new drugs (e.g., derivatives of impiridones [5]) in preclinical and clinical trials, radiotherapy remains the only standard of care currently available for patients with PED‐DHGG. However, most cases of PED‐DHGG, unlike adult GBM, do not show a survival benefit from combining temozolomide with radiation over radiation therapy alone [6]. Nevertheless, to date, radiation provides only temporary radiological improvement and short symptom relief, with a median overall survival of only 10–12 months [7, 8].

The diffuse hemispheric gliomas located in the hemispheric region and harboring the H3G34R/V mutation are less well‐studied than the diffuse midline gliomas. Depending on their accessibility, these hemispheric tumors are often treated with a combination of partial resection and radiation therapy. However, here too, the outcome of this treatment is poor, with a median overall survival of approximately 17.3 months [9]. Therefore, identifying new radiosensitizers and elucidating their downstream effectors is necessary to improve the outcome of radiotherapy in patients with these tumors. We have previously demonstrated that the two inhibitors of PeIF2α phosphatase—salubrinal and raphin‐1—decrease the survival of PED‐DHGG—derived cell lines and increase the sensitivity of these cells to antineoplastic treatments. Also, our experiments with the phosphomimetic [S51D] eIF2α variant further confirmed that increase in PeIF2α decrease PED‐DHGG survival and participate in modulating their response to PARP‐1 inhibitors [10].

The PeIF2α phosphatase is composed of a protein phosphatase‐1 catalytic subunit (PP1c) in combination with one of two possible regulatory subunits—either CReP or the stress‐induced GADD34 (growth arrest and DNA damage‐inducible 34) protein—which, together with G‐actin, control the activity and specificity of the PeIF2α phosphatase [11, 12]. Boyce et al. reported that salubrinal inhibits PeIF2α phosphatase activity by interfering with the interactions between PP1c and either one of these two regulators [13]. However, later studies showed that salubrinal does not interfere with the binding of GADD34 to either PP1c or PeIF2α, and at higher concentrations, it even strengthens the association between GADD34 and PP1c [14]. It also has a milder inhibitory effect on the holophosphatase *in vitro* than within the cells [15]. In addition, salubrinal does not increase the phosphorylation of histone 3 [13], indicating that it does not inhibit the activity of protein phosphatase 2. However, its mode of action toward PeIF2α phosphatase has yet to be defined [14, 15].

Another PeIF2α inhibitor, raphin‐1, was recently synthesized by Krzyzosiak et al. [16]. Unlike salubrinal, raphin‐1 is highly soluble and orally available and does not affect long‐term memory in mice. Moreover, in contrast to salubrinal, the direct interaction of raphin‐1 with CReP and its effect on CReP conformation have been demonstrated through protease protection and direct binding assays, which showed that raphin‐1 interferes with the binding of PeIF2α to CReP. Raphin‐1 was also shown to induce a valosin‐containing protein (VCP/p97)‐dependent degradation of CReP, as a further indication that it alters CReP conformation [16]. The affinity of raphin‐1 toward CReP‐PP1, which is 33‐fold stronger than its affinity toward GADD34‐PP1, was evaluated using an *in vitro* plasmon assay. However, it was calculated that while, at 10 μm, it engages all CReP molecules in HeLa cells, at higher concentrations, it also inhibited the activity of GADD34‐PP1 in these cells [16]. In contrast to raphin‐1, it has been suggested that guanabenz and sephin‐1 inhibit the GADD34‐PP1c complex [17, 18]. Nevertheless, later studies argued that these compounds do not affect the activity of GADD34‐PP1c *in vitro* or delay the *in situ* dephosphorylation of PeIF2α in kinase shut‐off experiments [15]. Thus, we did not include them in our experiments.

Subsequent studies have demonstrated that both GADD34 and CReP perform cellular functions other than regulating PeIF2α phosphatase. For instance, Krokowski et al. [19] showed that inhibition of the GADD34‐PP1 complex in corneal cells during hyperosmotic stress alters the microtubule network, leading to Golgi fragmentation and interference with the processing and trafficking of proteins from the Golgi to the plasma membrane. These GADD34‐PP1‐regulated processes were independent of its interaction with PeIF2α [19]. Kloft et al. also showed that CReP interacts with PeIF2α to regulate vesicular traffic in a PP1‐independent manner [20]. Notably, Kastan et al. demonstrated that in HeLa cells transfected with poliovirus, CReP maintains the association of the translational machinery with the endoplasmic reticulum (ER) and mediates viral protein translation that is immune to increased PeIF2α in the cytosol. Interestingly, an shRNA‐dependent decrease in CReP expression inhibits both the translation of the viral proteins and the translation of BiP, a chaperone belonging to the heat shock protein 70 family, which is required for the survival of cells in general and cancer cells in particular [21, 22].

Experiments with GADD34 knockout mice have yielded live mice with deficits in hemoglobin synthesis and metabolic dysregulation [23]. In contrast, abrogating CReP expression yielded pups that died shortly after birth, while GADD34/CReP double‐knockout mice failed to develop embryos altogether [24]. These experiments, like the ones cited above, suggest that GADD34 and CReP fulfill overlapping and different roles.

Therefore, it follows that the different modes of interaction of salubrinal and raphin‐1 with CReP and GADD34 may translate into different quantitative or time‐dependent effects on their shared targets or into different downstream effectors altogether. Indeed, our results show that although both raphin‐1 and salubrinal increase the cellular levels of PeIF2α, they differ in their effects on the expression of CReP, BiP, spliced x‐box binding protein‐1 (XBP1s), caseinolytic protease X (ClpX), and DR5 suggesting that PeIF2α independent processes are involved in mediating the effect of these drugs on PED‐DHGG survival. Therefore, the aim of this study was to identify both their PeIF2α‐phosphatase‐dependent and PeIF2α‐phosphatase‐independent molecular targets, as this is relevant to the development of drugs for treating PED‐DHGG.

## 2 Materials and methods

### 2.1 Cell lines

SU‐DIPG‐VI‐GFP‐LUC (SU‐DIPG‐VI) (RRID: CVCL_IT40) cell line was a generous gift from Dr Michelle Monje (Stanford University, Stanford, CA). KNS‐42 (RRID: CVCL_0378) cell line was obtained in 2019 from the Japanese Collection of Research Bioresources Cell Bank (Osaka, Japan). The short tandem repeat profile of these cell lines has recently been authenticated at the Technion Genomics Center (Haifa). Wild‐type mouse embryonic fibroblasts (MEF^WT^) and MEF cells homozygous for [S51A] eIF2α mutation (MEF^S51A^) cell lines [25] were a kind gift from Dr Randal J. Kaufman (Sanford Burnham Prebys, San Diego, CA, USA). Following a short propagation period, all cell lines were frozen, and aliquots were resuscitated and used for about 10 weeks. All cells were routinely tested for mycoplasma.

### 2.2 Growth conditions

KNS‐42 cells were grown in Eagle's minimal essential medium supplemented with 5% fetal bovine serum (FBS), 1% l‐glutamine, and 1% penicillin–streptomycin (Biological Industries, Kibbutz Beit‐Haemek, Israel). SU‐DIPG‐VI cells were grown in tumor stem medium consisting of 50% neurobasal‐A medium, 50% DMEM/F‐12, 1% HEPES (1 m), 1% sodium pyruvate, 1% nonessential amino acids, 1% GlutaMAX, 1% antibiotic antimycotic, 2% B‐27 (Thermo Fisher Scientific, Waltham, MA, USA), 0.02% heparin (STEMCELL Technologies, Vancouver, Canada), 20 ng·mL^−1^ EGF and bFGF, and 10 ng·mL^−1^ PDGF‐AA and PDGF‐BB (PeproTech Asia, Rehovot, Israel). MEF cells were grown in DMEM containing 10% FBS, 1% l‐glutamine, 1% penicillin–streptomycin, 1% MEM Non‐Essential Amino Acids n (Thermo Fisher Scientific) and 2% MEM Amino Acids (Merck KGaA, Darmstadt, Germany).

### 2.3 Reagents

Raphin‐1 (6760) was obtained from Tocris Bioscience (Bristol, UK). Salubrinal (#HY‐15486), CB‐5083 (#HY‐12861), and HA15 (#HY‐100437) were from MedChem Express (NJ). ONC‐201 (TIC10) (S7963) was purchased from Selleckchem (Houston, TX, USA). Silencer® Negative Control siRNA #2 (siCON, Ambion Inc. Austin, TX, USA) was obtained from Ambion (#AM4613, Thermo Fisher Scientific) and CReP siRNA (siCReP, #sc‐88234) was from Santa Cruz Biotechnology (Dallas, TX, USA). HA (hemagglutinin)‐tagged WT, S51A, and S51D heIF2α in pcDNA3.1(+)‐N‐HA were obtained from GenScript USA Inc. (Piscataway, NJ, USA). Integrated stress response inhibitor (ISRIB) (#SML0843) and cycloheximide (CHI) (#C1988) were from Sigma‐Aldrich (Merck KGaA). Z‐VAD‐FMK (A1902) was purchased from APExBIO (Houston, TX, USA). All drugs were added from stock solutions in dimethyl sulfoxide (DMSO) and control cultures received an equal amount of the vehicle. The final concentration of DMSO in the culture medium did not exceed 0.05%.

### 2.4 Survival assays

SU‐DIPG‐VI cells were plated in 96‐well plates, and KNS‐42 cells were plated in triplicates in 6‐well plates, at densities of 10 000 and 40 000 cells per well, respectively. Twenty‐four hours later, cells from one triplicate were dissociated and counted to determine cell number at *T*_0_. At that time, HA15 or ONC201 was added at the indicated concentrations. MEF^WT^ and MEF^S51A^ cells were plated in 6‐well plates at a density of 50 000 cells per well. Twenty‐four hours later, cells from one triplicate were dissociated and counted to determine cell number at *T*0, and raphin‐1 was added. Cells were dissociated 72 h following drugs addition and trypan‐blue‐excluding cells (*T*_e_) from each triplicate were counted either by manual counting, while observing the cells under a microscope, or by subjecting them to automated counting (Countess II FL Automated Cell Counter, Thermo Fisher Scientific). Both methods yielded similar results. To determine the effect of raphin‐1 on the cells' sensitivity to irradiation, SU‐DIPG‐VI cells were plated in 96‐well plates at a density of 4000 cells per well and KNS‐42 cells were plated for clonogenic assay, as described in Section 2.5. Twenty‐four hours later, cells from one triplicate were dissociated and counted to determine cell number at *T*_0_. At that time, the cells were irradiated, and raphin‐1 was added at the indicated concentrations. Following incubation for 7 days, the cells were dissociated, and trypan‐blue‐excluding cells from each triplicate were counted (*T*_e_).

Cell survival (%) was calculated according to the following formula:

$$100\times \frac{\left(\mathrm{AVG}\;\text{cell}\;\#\;\mathrm{at}\;T_{e}\;\left(\text{treated}\right)-\mathrm{AVG}\;\text{cell}\;\#\;\mathrm{at}\;T_{0}\right)/\mathrm{AVG}\;\text{cell}\;\#\;\mathrm{at}\;T_{0}}{\left(\mathrm{AVG}\;\text{cell}\;\#\;\mathrm{at}\;T_{e}\;\left(\text{control}\right)-\mathrm{AVG}\;\text{cell}\;\#\;\mathrm{at}\;T_{0}\right)/\mathrm{AVG}\;\text{cell}\;\#\;\mathrm{at}\;T_{0}}.$$

Cell death (%) was calculated as follows:

$$100\times \left(\left(\mathrm{AVG}\;\text{cell}\;\#\;\mathrm{at}\;T_{e}-\mathrm{AVG}\;\text{cell}\;\#\;\mathrm{at}\;T_{0}\right)/\left(\mathrm{AVG}\;\text{cell}\;\#\;\mathrm{at}\;T_{0}\right)\right)$$

All experiments were conducted in triplicates.

### 2.5 Clonogenic survival assay

KNS‐42 cells (500 per well) were plated in triplicates in 6‐well plates. Twenty‐four hour post‐plating, cells were irradiated and raphin‐1 was added. When 90–95% of the colonies in the control plate possessed more than 50 cells (14 days post‐plating), colonies were fixed with 70% ethanol and stained with 0.05% crystal violet (Merck KGaA) before counting.

### 2.6 Radiation

The cells were irradiated in an X‐ray irradiator (Polaris sc‐500 series II) at a dose rate of 100 cGy·min^−1^.

### 2.7 Cell lysis and western blotting

SU‐DIPG‐VI cells were plated at a density of 2 × 10^5^ cells·mL^−1^ in T25 flasks, and KNS‐42 cells were plated at a density of 24 000 cells·cm^−2^ in 60‐mm plates. Drugs were added and/or irradiation was administered 24 h after plating, and incubation was continued for the specified time before the cells were harvested, washed with ice‐cold DPBS, and collected in buffer containing 150 mm of NaCl, 50 mm of Tris (pH 7.5), 1.7 mm of EDTA, 1.5 mm of sodium orthovanadate, 100 mm of sodium fluoride, 1.5 μg·mL^−1^ of pepstatin, 2% SDS, and Roche protease inhibitor cocktail. Following heating at 95 °C and clearing by centrifugation, the protein content was determined with a bicinchoninic acid reagent (Bio‐Rad, Hercules, CA, USA). All gels included molecular weight markers (Thermo Scientific™ #26616). Gels were blotted onto nitrocellulose membranes, and treatment‐induced changes in protein level were determined by cutting horizontal strips at the appropriate molecular weight and incubating them with the relevant antibodies. Following chemiluminescence detection, the relative positions of the proteins of interest and their nearest molecular weight marker were determined by aligning the films with the relevant membrane strip. Chemiluminescence detection was performed using West Pico ECL substrate (Thermo Fisher Scientific), with imaging conducted through X‐ray film exposure or digital capture using either the Bio‐Rad ChemiDoc MP or the Invitrogen iBright™ Imaging systems. Values for the integrated light density from several experiments were obtained with imagej software (Version 1.53e, National Institute of Health, Bethesda, MD, USA) and used to verify the significance (*P* < 0.05) of the difference between control and treated cells. To ensure equal loading, blots from a twin run were stained with Ponceau‐S, and the intensity of the image of each vertical strip was quantified by using imagej software.

Rabbit anti‐PeIF2α (9721), anti‐eIF2α (#9722), anti‐BiP (#3177), anti‐CHOP (#5554), anti‐cleaved caspase‐8 (#9496), anti‐DR5 (#8074), and anti‐XBP1s (#40435) were from Cell Signaling Technology (Danvers, MA, USA). Rabbit anti‐PPP1R15B (CReP) (#14634‐1‐AP) and anti‐GADD34 (#10449‐1‐AP) were from Proteintech (Rosemont, IL, USA). Rabbit anti‐ClpX (ab168338) and anti‐ATF4 (ab184909) were from Abcam (Cambridge, UK). HRP‐coupled goat anti‐rabbit IgG was obtained from Jackson Immunoresearch Laboratories (111‐035‐144, West Grove, PA, USA).

### 2.8 Transfection with siRNA or DNA plasmids

One million SU‐DIPG‐VI cells were plated in 60 mm plates containing 5 mL of antibiotic‐free medium. Twenty‐four hours later, siRNA CReP or controls‐lipid complexes prepared in Opti‐MEM™ I Reduced Serum Medium, and the cells were transfected in the presence of Lipofectamine™ RNAiMAX Transfection Reagent (Thermo Fisher Scientific) for 24 h, according to the manufacturer's instructions KNS‐42 were plated in six‐well plates for survival assay at a density of 13 200 cells·cm^−2^ and transfected 24‐h post‐plating with HA‐tagged eIF2a variants in pcDNA3.1(+)‐N‐HA by employing 1 μg·mL^−1^ plasmids and jetPEI transfection reagent (Polyplus, New York, NY, USA), according to the manufacturer's instructions.

### 2.9 RNA extraction, RT‐PCR, and real‐time PCR

SU‐DIPG‐VI and KNS‐42 cells were plated at a density of 2 × 10^5^·mL^−1^ in T25 flasks and at 24 000 cells·cm^−2^ in 60‐mm plates, respectively. The drugs were added 24 h later. At the indicated time, RNA was extracted using Direct‐zol RNA Miniprep (Zymo Research, Irvine, CA, USA). RT‐PCR was carried out with 1 μg of RNA using the High‐Capacity cDNA Reverse Transcription Kit (Applied Biosystems, Foster City, CA, USA) according to the manufacturer's instructions. Real‐time PCR was performed with 2xqPCRBIO SyGreen Blue Mix (PCR Biosystems, London, UK), according to the manufacturer's instructions and carried out using a StepOnePlus™ system (Applied Biosystems) with the following primers:

hCReP F: 5′‐AGCGTGACGTTCTTTCTGGA‐3′,

R: 5′‐CCATGGTCCTTTGCGATCCT‐3′.

hBiP (HSPA5) – F: 5′‐GAACGTCTGATTGGCGATGC‐3′,

R: 5′‐ACCACCTTGAACGGCAAGAA‐3′.

hGADD34 F: 5′‐AGCTAGGACTCCTCTGGCAA‐3′,

R: 5′‐GCTTCAGGAAGGGAACTGCT‐3′.

hDR5 F: 5′‐GGGAGCCGCTCATGAGGAAGTTGG‐3′,

R: 5′‐GGCAAGTCTCTCTCCCAGCGTCTC‐3′.

hXBP1s F: 5′‐GCTGAGTCCGCAGCAGGT‐3′,

R: 5′‐CTGGGTCCAAGTTGTCCAGAAT‐3′.

hActin F: 5′‐CACCATTGGCAATGAGCGGTTC‐3′,

R: 5′‐AGGTCTTTGCGGATGTCCACGT‐3′.

hGAPDH F: 5′‐GTCTCCTCTGACTTCAACAGCG‐3′,

R: 5′‐ACCACCCTGTTGCTGTAGCCAA‐3′.

Glyceraldehyde‐3‐phosphate dehydrogenase or β‐actin were used as housekeeping genes, and differences in the mRNA levels of treated and control cells were evaluated by employing the ΔΔ*C*_t_ method.

### 2.10 Electron microscopy

Following 48 h incubation with raphin‐1, cells were washed twice with DPBS and fixed for 1 h with formaldehyde/glutaraldehyde 2.5% in 0.1 m sodium cacodylate buffer at a pH of 7.4 (Electron Microscopy Sciences, Hatfield, PA, USA). Thin sections were viewed with a TEI Tecnai™ Spirit transmission electron microscope.

### 2.11 Statistical analysis

The significance of the differences between treated and control groups was verified using one‐sample *t*‐test or unpaired Student's *t*‐test. The level of statistical significance in each comparison was set at *P* ≤ 0.05.

## 3 Results

### 3.1 The effect of raphin‐1 and salubrinal on the cellular levels of CReP and BiP in PED‐DHGG

**Fig. 1:**
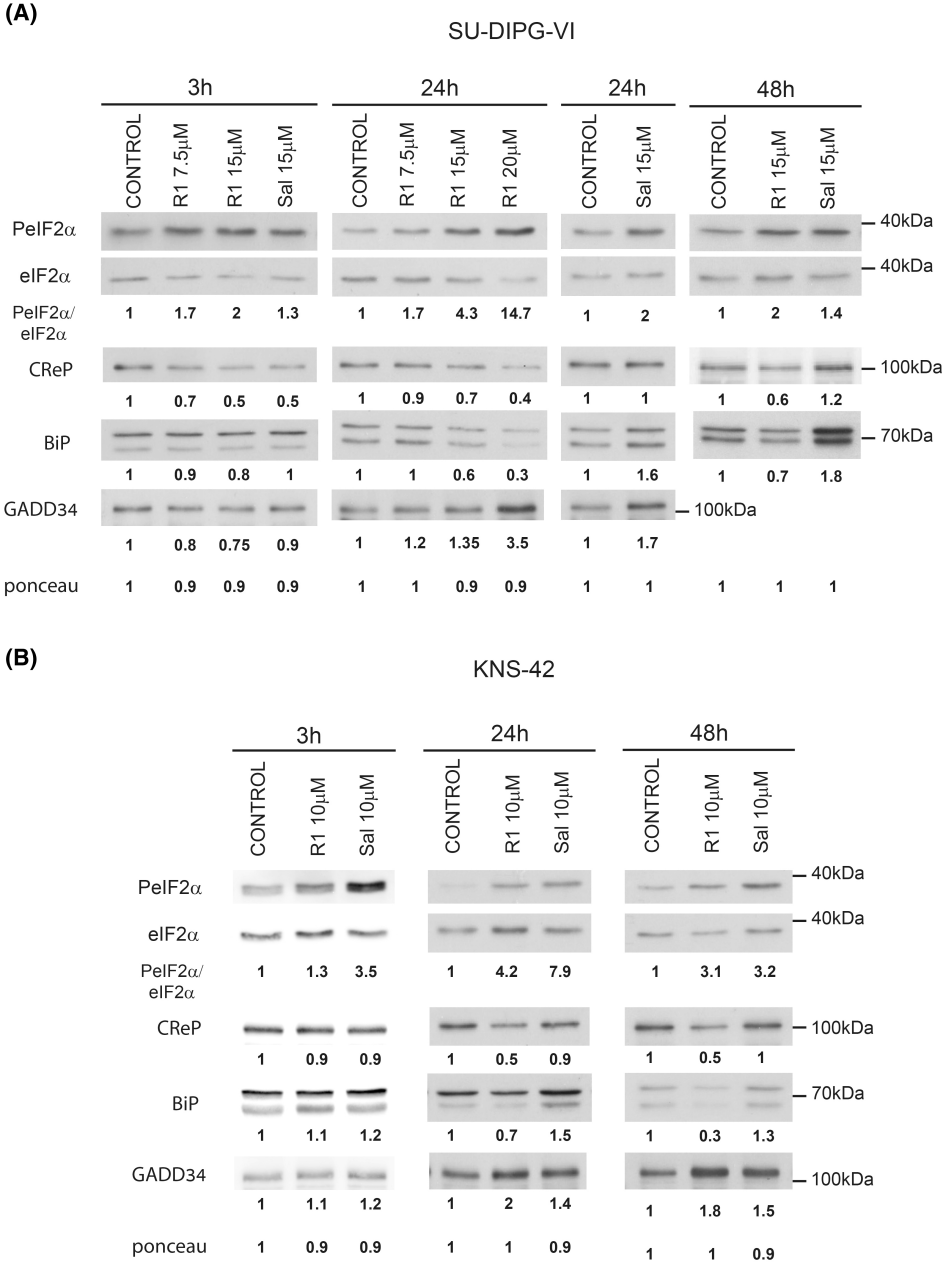
Raphin‐1 but not salubrinal leads to a sustained decrease in the cellular level of CReP and BiP. SU‐DIPG‐VI and KNS‐42 cells (A and B, respectively) were treated with the specified concentrations of either raphin‐1 or salubrinal for the indicated time and processed for western blot analysis as described in Section 2. Numbers at the bottom of the autoradiograms indicate treatment‐dependent changes in the level of the proteins and equal loading control (Ponceau). The experiments were reproduced several times with similar results. The number of determinations for each experimental condition was as follows: SU‐DIPG‐VI – 3 h: R1 *n* = 7, Sal *n* = 3, 24 h: R1 CReP *n* = 14, BiP *n* = 13, Sal *n* = 5, 48 h: R1 *n* = 8, Sal *n* = 5; KNS‐42 – 3 h: *n* = 2, 24 h: *n* = 4, 48 h: *n* = 2. R1‐raphin‐1, Sal‐salubrinal.

**Fig. 2:**
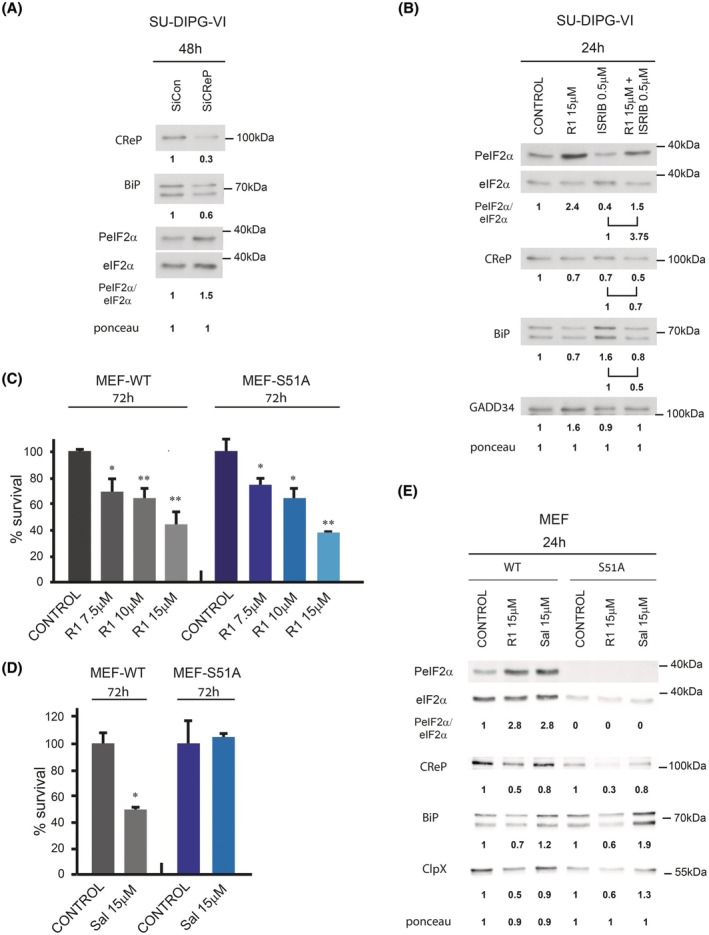
Raphin‐1 decreases CReP and BiP levels and cell survival via PeIF2α‐independent manner. (A) Transfection with CReP siRNA is associated with decreased BiP level. (B) The effect of ISRIB on the level of CReP and BiP in raphin‐1‐treated cells. (E) the effect of raphin‐1 and salubrinal on protein expression in MEF‐WT and MEF‐S51A‐eIF2a. (A, B, E) SU‐DIPG‐VI and MEF cells were treated with the indicated concentrations of either raphin‐1 or salubrinal for the indicated time and processed for western blot analysis as described in Section 2. Numbers at the bottom of the autoradiograms indicate treatment‐dependent changes in the level of the proteins and equal loading control (Ponceau). The number of determinations for each experimental condition were as follows: A: *n* = 3, B: *n* = 3, E: *n* = 2. R1‐raphin‐1, Sal—salubrinal. (C, D) The effect of raphin‐1 and salubrinal on the survival of MEF‐WT and MEF‐S51A‐eIF2a. MEF cells were plated in triplicates and treated with raphin‐1 and salubrinal as described in Section 2. Values are mean survival (%) ± SD. The significance of the differences between treatments and control were determined using an unpaired Student *t*‐test – *P < 0.05, **P < 0.005.

Our experiments included two cell lines derived from PED‐DHGG: KNS‐42, which harbors the H3.3G34V mutation, and SU‐DIPG‐VI, which harbors the H3.3K27M mutation. Given the reported effects of raphin‐1 on CReP levels and conformation [16] and the role of CReP in ER‐associated BiP translation during viral transfection [22], we compared the effects of raphin‐1 versus salubrinal on the cellular levels of these two proteins. In SU‐DIPG‐VI (Fig. 1A), at 3 h, treatment with either drug increased PeIF2α and decreased CReP, whereas only raphin‐1 treatment led to a small but reproducible decrease in BiP (20% ± 8, *n* = 7; *P* < 0.001). At 3 h, neither drug had increased the level of GADD34, a known downstream effector of increased PeIF2α. At 24 h after drug addition, the sustained effect of either raphin‐1 or salubrinal on PeIF2α was associated with an increased expression of GADD34 (Figs 1A and 2B). In addition, while the effect of raphin‐1 on CReP level was attenuated at 7.5 μm, it was clearly visible at 15 μm, where it led to a 33% ± 13 reduction (*n* = 14; *P* < 0.001) in CReP level, which lasted throughout the 48‐h experimental period. Conversely, the effect of salubrinal on CReP dissipated in a time‐dependent manner and disappeared altogether by 48 h after treatment initiation. By 24 h, raphin‐1 led to a 53% ± 16 (*n* = 13; *P* < 0.001) reduction in BiP, while salubrinal gradually increased the level of BiP (Fig. 1A).

Similar differences between the effects of salubrinal and raphin‐1 on the levels of CReP and BiP were noted in the KNS‐42 cells. Because KNS‐42 is more sensitive than SU‐DIPG‐VI to raphin‐1 [10], we conducted these experiments with lower concentrations of raphin‐1 and salubrinal. Thus, at 10 μm, both drugs increased the levels of PeIF2α and GADD34 by 24 h, but only raphin‐1 induced decreases in CReP and BiP. Raphin‐1 decreased CReP by 37% ± 11 (*n* = 4; *P* < 0.001) and BiP by 30% ± 11 (*n* = 4; *P* < 0.001). Salubrinal did not lead to any significant change in the level of CReP, but it did increase BiP by 66% ± 27 (*n* = 4; *P* < 0.001) (Fig. 1B). Note, however, that the level of BiP was significantly higher in the KNS‐42 cells than in the SU‐DIPG‐VI cells (Fig. S1A), suggesting that KNS‐42 cells may be less sensitive than SU‐DIPG‐VI cells to a similar fractional decrease in BiP.

These results showed that despite their stimulation of a similar increase in PeIF2α, raphin‐1 and salubrinal exhibited different time‐dependent and qualitative effects on the cellular levels of CReP and BiP. In addition, increasing PeIF2α or GADD34 was clearly insufficient for decreasing the level of CReP or BiP. Rather, the increased level of GADD34‐PP1c may have competed with the binding of salubrinal to CReP‐PP1c—much more so than with the binding of raphin‐1 to that complex—thus leading to the observed differences between the two drugs. In addition, a 3‐h incubation of SU‐DIPG‐VI cells with salubrinal decreased the level of CReP, with no noticeable effect on BiP. However, raphin‐1‐treated cells showed an association between decreases in CReP and decreased BiP, which may reflect the effect of raphin‐1 on both CReP level and conformation (Fig. 1A).

### 3.2 Raphin‐1 can decrease CReP and BiP protein levels and cell survival in a PeIF2α‐independent manner

Transfection with CReP siRNA, like raphin‐1 treatment, also increased PeIF2α and decreased BiP by 42% ± 6 (*n* = 3; *P* < 0.001) (Fig. 2A). We evaluated the extent to which increased PeIF2α mediated the effect of raphin‐1 on the levels of CReP and BiP by examining the effect of the integrated stress response inhibitor (ISRIB) [26]. PeIF2α attenuates global protein translation by inhibiting the GDP/GTP exchange activity of eIF2B and, as a result, the replenishment of eIF2·GTP from eIF2·GDP. ISRIB and PeIF2α bind to eIF2B at different sites in a mutually exclusive manner [27], such that the binding of ISRIB counteracts the inhibitory effect of PeIF2α on the exchange activity of eIF2B and its consequences. In our experiments, ISRIB decreased PeIF2α and, as expected, decreased the PeIF2α‐mediated increase in GADD34 expression. Interestingly, when used on its own, ISRIB decreased CReP and increased BiP, suggesting that the basal level of PeIF2α activity supports the expression of CReP but diminishes the expression of BiP. Nevertheless, ISRIB did not abrogate the effect of raphin‐1 on the level of either CReP or BiP (Fig. 2B). This result shows that the decreased CReP and BiP levels in raphin‐1‐treated cells do not result directly or indirectly from a PeIF2α‐dependent modulation of protein translation.

We then determined the effects of both raphin‐1 and salubrinal on the levels of CReP and BiP in MEF^WT^ and MEF^S51A^. We observed that the different effects of raphin‐1 and salubrinal on the levels of CReP and BiP in PED‐DHGG were maintained in MEF^WT^ and MEF^S51A^ (Fig. 2E). The effect of raphin‐1 on the CReP level exceeded that of salubrinal, and whereas raphin‐1 decreased the level of BiP, salubrinal treatment increased it. This experiment indicated that the decreased levels of CReP and BiP in raphin‐1‐treated cells and the increased level of BiP in salubrinal‐treated cells were independent of increases in PeIF2α.

As we have reported previously [10], and as noted in Fig. S2, the expression of the phosphomimetic [S51D] eIF2α variant showed decreased survival of PED‐DHGG similar to the effects observed with raphin‐1 and salubrinal treatment. This indicated that increased PeIF2α participates in mediating the physiological effects of the drugs. However, while both drugs decreased the survival of MEF^WT^ cells, only raphin‐1 affected the survival of MEF^S51A^ cells (Fig. 2C,D). These experiments demonstrated that raphin‐1 could affect cell survival via PeIF2α‐independent processes.

### 3.3 Mechanisms that underlie the effects of raphin‐1 and salubrinal on the expression of CReP and BiP

**Fig. 3:**
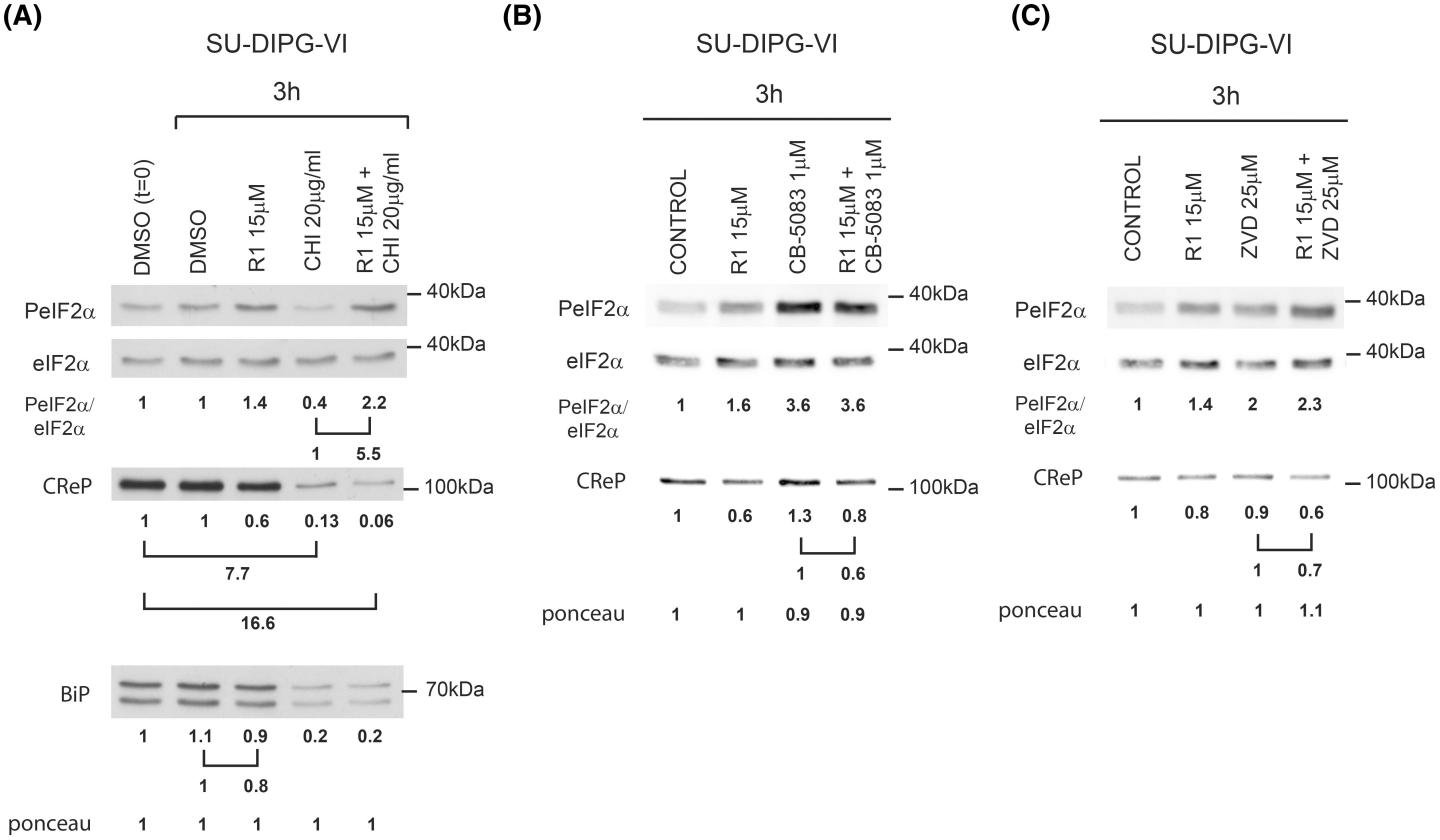
Mechanisms that underlie the effect of raphin‐1 on CReP and BiP level. SU‐DIPG‐VI cells were treated with the specified concentrations of drugs and for the indicated time, then processed for western blot analysis as described in Section 2. Numbers at the bottom of the autoradiograms indicate treatment‐dependent changes in the level of the proteins and equal loading control (Ponceau). The number of determinations for each experimental condition was as follows: A: *n* = 2, B: *n* = 2, C: *n* = 2. R1‐raphin‐1, CHI—cycloheximide, CB‐5083—VCP/P97 inhibitor, ZVD—pan‐caspase inhibitor z‐vad‐fmk.

Analysis of CReP and BiP mRNA levels at 3, 12, and 24 h following the addition of salubrinal and raphin‐1 did not provide an explanation for the effect of the two drugs on the levels of CReP and BiP proteins (Fig. S3). Therefore, to determine whether raphin‐1 increases the degradation rate of CReP (known for its short half‐life [16, 22]), we employed cycloheximide (CHI). Cotreatment with CHI and raphin‐1 for 3 h showed that while CReP translation occurs continuously in the presence of raphin‐1, the rate of its degradation was about twofold higher in the presence of the drug compared to untreated control cells (Fig. 3A). The half‐life of BiP was also short, as revealed by its rapid decline in the presence of CHI [22]. However, in contrast to its effect on CReP, raphin‐1 did not accelerate the degradation of BiP, suggesting that it reduced the BiP level by attenuating BiP translation (Fig. 3A). This result is consistent with the findings of Kastan et al. [22], who showed that CReP, through its interaction with eIF2α, maintains the association of the translation initiation machinery with the ER and mediates the ER‐associated translation of BiP. Thus, the effect of raphin‐1 on both the CReP level and conformation was expected to have a negative effect on BiP translation. Notably, in contrast to its effects on CReP and BiP, the addition of CHI did not alter the level of eIF2α during the experimental period, suggesting that eIF2α has a longer half‐life than CReP or BiP.

Treating HEK293 and HeLa cells with the VCP/p97 inhibitor CB‐5083 increased CReP in both control and ultraviolet‐irradiated cells, demonstrating its participation in CReP turnover [28]. Krzyzosiak et al. concluded that in HeLa cells, raphin‐1 leads to a VCP/p97‐dependent degradation of CReP [16]. However, the inclusion of CB‐5083 in our experiments did not abrogate the effect of raphin‐1 on CReP (Fig. 3B). Furthermore, treatment with the pan‐caspase inhibitor z‐vad‐fmk did not alter the effect of raphin‐1 on CReP (Fig. 3C). Hence, the specific degradative system activated by raphin‐1 has yet to be identified.

### 3.4 Raphin‐1 and salubrinal differ in their effects on the unfolded protein response (UPR)

BiP is a chaperone that assists with the folding of newly synthesized peptides and their transport into different cellular organelles, as well as with the targeting of misfolded peptides to degradative systems [21]. Via its interaction with peptide lumenal domains, BiP also suppresses the activation of the three sensors of ER stress: PKR‐like ER kinase, inositol‐requiring enzyme 1 (IRE1α), and activating transcription factor 6. Thus, a decrease in BiP can contribute to both increased ER overload and the activation of UPR. While the initial activation of UPR leads to a corrective response, sustained stress leads to terminal UPR and cell death [29, 30].

We therefore compared the effects of raphin‐1 and salubrinal on the levels of spliced XBP1 (XBP1s) and DR5, which are indicative of UPR activation. Increases in XBP1s mRNA reflect the noncanonical regulated IRE1‐dependent decay (RIDD) activity of IRE1α, which involves the splicing and ligation of XBP1 mRNA [29]. Unresolved stress can also lead to IRE1α‐dependent activation of the Jun N‐terminal kinase (JNK) pathway and an increase in DR5, which is associated with activation of caspase‐8 and cell death [31, 32].

**Fig. 4:**
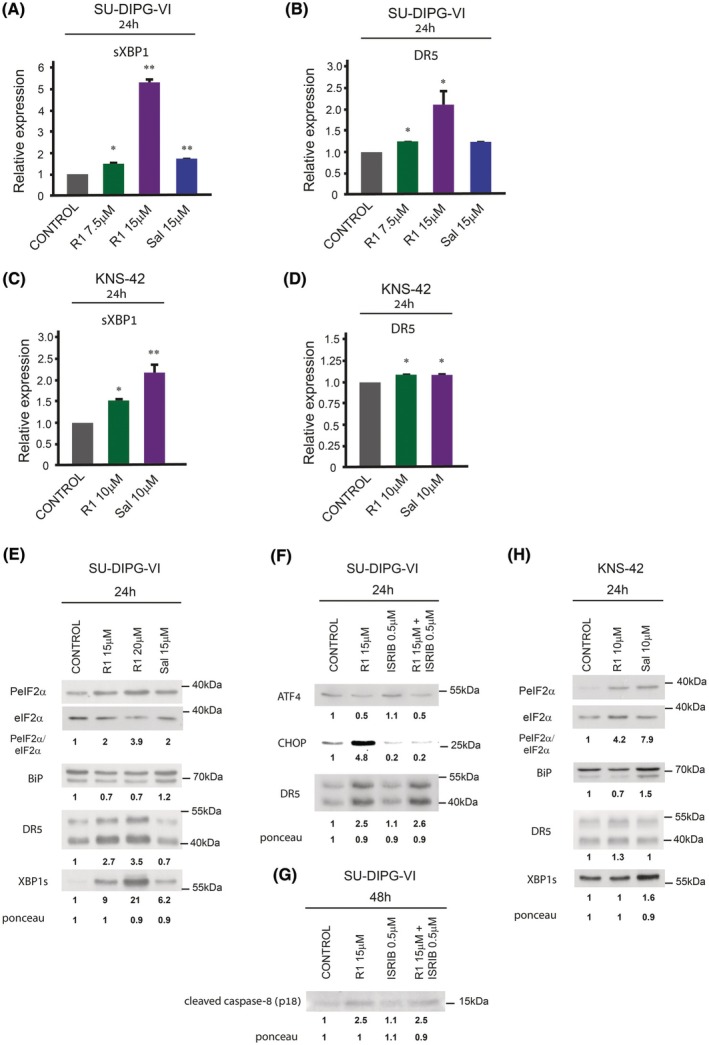
Raphin‐1 and salubrinal differ in their effect on UPR downstream effectors. (A–D) SU‐DIPG‐VI and KNS‐42 cells were treated with the indicated concentrations of raphin‐1 and salubrinal for 24 h. RNA was extracted and treatment‐induced changes in DR5 and XBP1s mRNA were evaluated by qRT‐PCR as described in Section 2. Data are mean relative quantification (RQ) ± SD of two independent experiments. The significance of the differences between treatments and control was determined using an unpaired Student *t*‐test—*P < 0.05, **P < 0.005. (E–G) SU‐DIPG‐VI and KNS‐42 cells were treated with the specified concentrations of drugs and for the indicated time and processed for western blot analysis as described in Section 2. Numbers at the bottom of the autoradiograms indicate treatment‐dependent changes in the level of the proteins and equal loading control (Ponceau). The number of determinations for each experimental condition was as follows: E: DR5 *n* = 5, XBP1s *n* = 2, F: *n* = 4, G: *n* = 2, H: *n* = 3. R1‐raphin‐1, Sal‐salubrinal.

We found that in the SU‐DIPG‐VI cells, the effect of salubrinal on the XBP1s protein level preceded that of raphin‐1 and was already noticeable at 3 h after treatment initiation (Fig. S1B). However, by 24 h, raphin‐1 treatment led to a higher level of XBP1s mRNA and protein than that obtained with salubrinal (Fig. 4A,E). However, the two drugs differed sharply in their effects on the expression of DR5. While raphin‐1 increased the levels of DR5 mRNA and protein, salubrinal failed to do so (Fig. 4B,E).

PeIF2α is known to increase cellular levels of activating transcription factor 4 (ATF4), which then leads to increased levels of C/EBP homologous protein (CHOP)—an activator of DR5 gene transcription [33]. Our results showed that raphin‐1 treatment leads to a drastic ISIRB‐independent reduction in ATF4 but increases CHOP. Interestingly, our experiments demonstrated that while the addition of ISRIB abrogated the increase in the CHOP level, it did not affect the level of DR5, suggesting that the raphin‐1‐induced expression of DR5 is not dependent on this canonical downstream effector of PeIF2α (Fig. 4F). DR5 is known to decrease cell survival via the activation of caspase‐8 [31, 32]. Indeed, as shown in Fig. 4G, the increased level of DR5 in raphin‐1‐treated cells was associated with an ISRIB‐independent activation of caspase‐8, as reflected by the accumulation of the p18 cleavage form of pro‐caspase‐8 [34].

In KNS‐42 cells, as in SU‐DIPG‐VI cells, raphin‐1 increased the level of DR5 protein, whereas salubrinal did not (Fig. 4H). Interestingly, despite the differences in their effects on the DR5 protein, both drugs induced only a slight increase, if any at all, in the level of DR5 mRNA (Fig. 4D), suggesting enhanced translation or stability of the DR5 protein in raphin‐1 relative to salubrinal‐treated cells. Raphin‐1 treatment failed to increase the cellular level of XBP1s protein at either 3 h (Fig. S1C) or 24 h after treatment initiation (Fig. 4H). The small increase in XBP1s mRNA in raphin‐1‐treated cells did not materialize into increased XBP1s protein. This suggested that XBP1s mRNA, as with BIP mRNA, may be translated via ER‐associated machinery and therefore higher levels of XBP1s mRNA are required in order to bring about an increase in XBP1s protein in the presence of raphin‐1. By contrast, salubrinal treatment induced higher levels of both XBP1 mRNA and protein (Fig. 4C,H).

**Fig. 5:**
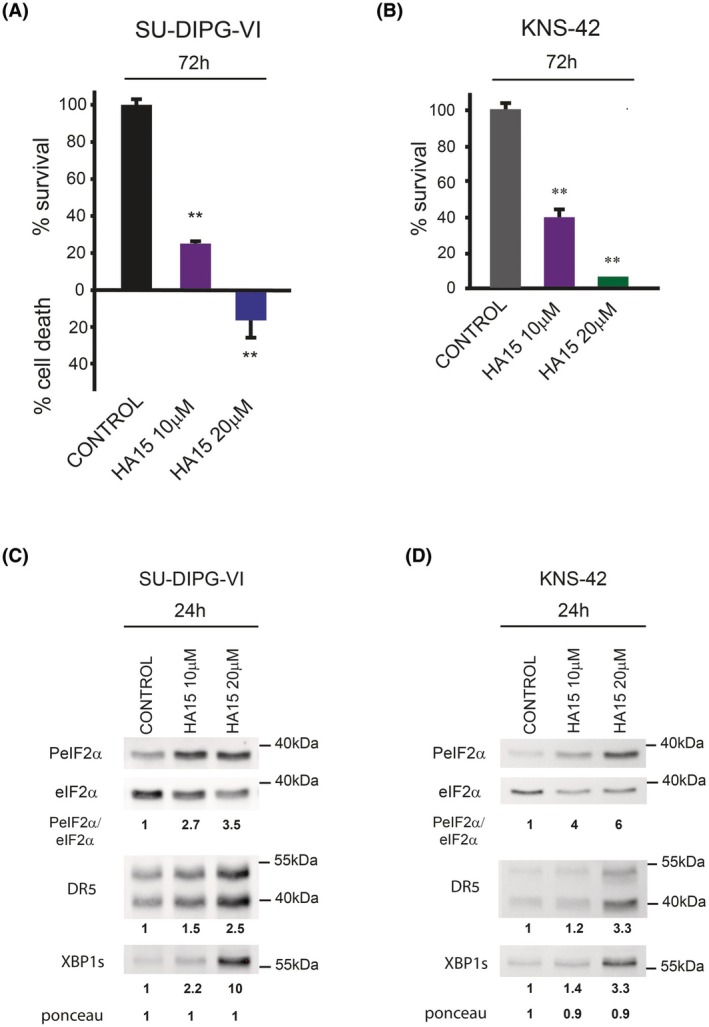
The effect of HA15, a specific inhibitor of BiP, on raphin‐1's downstream targets. (A, B) SU‐DIPG‐VI and KNS‐42 cells were plated in triplicates and treated with HA15 as described in Section 2. Values are mean survival (%) or cell death (%) ± SD of two independent experiments. The significance of the differences between treatments and control were determined using an unpaired Student *t*‐test—**P < 0.005. (C, D) SU‐DIPG‐VI and KNS‐42 cells were treated with the indicated concentrations of HA15 for 24 h and processed for western blot analysis as described in Section 2. Numbers at the bottom of the autoradiograms indicate treatment‐dependent changes in the level of the proteins and equal loading control (Ponceau). The number of determinations for each experimental condition were as follows: C: *n* = 2, D: *n* = 2.

We then examined whether the high level of BiP in KNS‐42 interfered with the activation of XBP1 expression by raphin‐1 by treating cells with HA15, a BiP‐specific inhibitor [35]. As shown in Fig. 5A,B, the survival of both cell lines was adversely affected by HA15 treatment. However, HA15 sensitivity was stronger in the SU‐DIPG‐VI cells than in the KNS‐42 cells. The addition of HA15 increased the XBP1s and DR5 levels in both cell types (Fig. 5C,D), suggesting that the high level of BiP in KNS‐42 cells interferes with the ability of raphin‐1 to activate IRE1α. Notably, salubrinal increased XBP1s without decreasing BiP at both 3 and 24 h in the SU‐DIPG‐VI cells (Fig. S1B, Figs 1A and 4E) and in KNS‐42 (Fig. S1C, Figs 1B and 4H). This suggested that, under our experimental conditions, salubrinal circumvented the inhibitory effect of BiP on the expression of XBP1s.

### 3.5 Raphin‐1 shares downstream effectors with ONC201

ONC201 is an imipridone, which is a compound that decreases the survival of cancer cells *in vitro* and in xenograft animal models [36, 37]. In clinical trials, although H3.3G34R/V‐mutated diffuse hemispheric gliomas have shown low sensitivity to ONC201 and its derivatives, this drug has shown promise in treating H3K27M‐mutated diffuse midline gliomas [38]. In various studies, sensitivity to ONC201 has been associated with different downstream effectors, including PeIF2α, ATF4, and DR5, as well as with the cellular levels of BiP [39, 40]. Recent studies have demonstrated that ONC201 binds and activates the mitochondrial serine protease caseinolytic peptide P (ClpP), while simultaneously decreasing the level of its chaperone, the ATP‐dependent ClpX, with cell death as a consequence [41]. Notably, cancer cells exhibit greater sensitivity than normal cells to the inhibition or overactivation of the ClpXP protease system [42, 43].

**Fig. 6:**
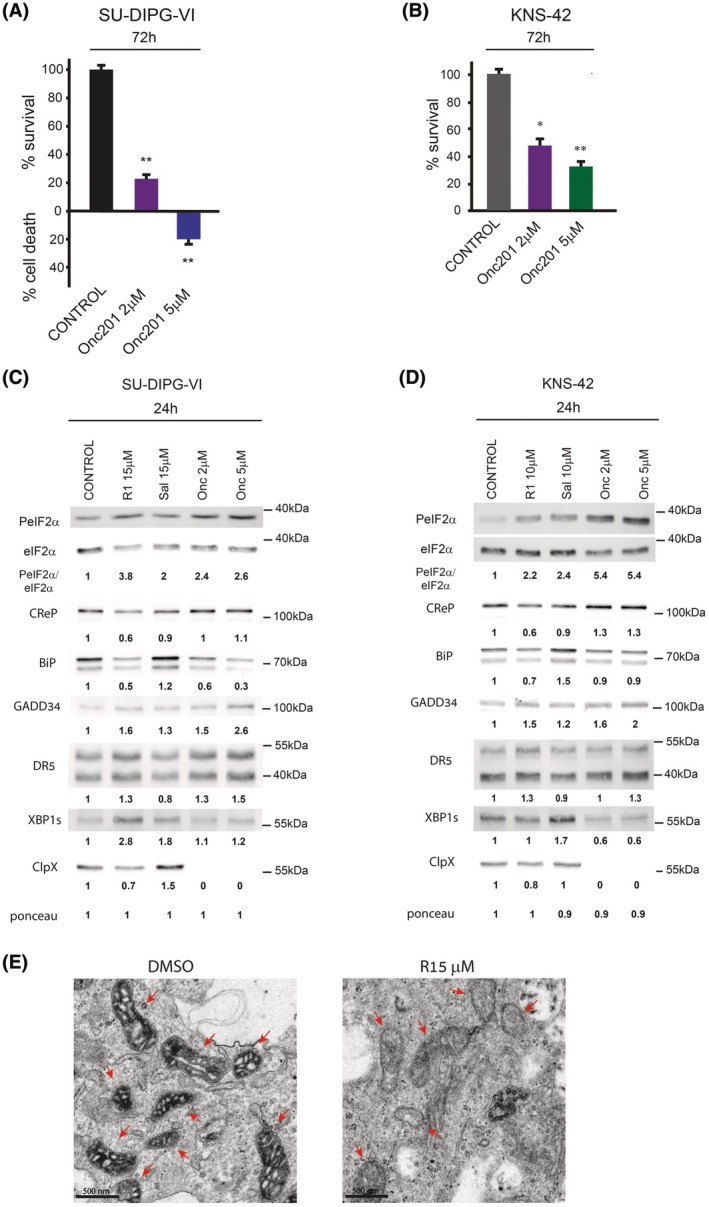
Raphin‐1 and ONC201 share downstream effectors in PED‐DHGG. (A, B) SU‐DIPG‐VI and KNS‐42 cells were plated in triplicates and treated with ONC201 as described in Section 2. Values are mean survival (%) or cell death (%) ± SD of two independent experiments. The significance of the differences between treatments and control was determined using an unpaired Student *t*‐test—*P < 0.05, **P < 0.005. (C, D) SU‐DIPG‐VI and KNS‐42 cells were treated with the indicated concentrations of raphin‐1, salubrinal, and ONC201 for 24 h and processed for western blot analysis as described in Section 2. Numbers at the bottom of the autoradiograms indicate treatment‐dependent changes in the level of the proteins and equal loading control (Ponceau). The number of determinations for each experimental condition was as follows: C: *n* = 3, D: *n* = 2. R1‐raphin‐1, Sal‐salubrinal, Onc‐ONC201. (E) SU‐DIPG‐VI cells were treated for 48 h with 15 μm raphin‐1, fixed, and processed for transmission electron microscopy, as described in Section 2. Bar = 500 nm, arrows point to mitochondria in control and raphin‐1‐treated cells. In control untreated cells, 20% of 400 counted mitochondria exhibited a decrease in their content, compared with 90% of 215 counted mitochondria in raphin‐1‐treated cells.

Given that the effect of raphin‐1 on PED‐DHGG survival was apparently associated with increased eIF2α phosphorylation, increased DR5, and reduced BiP, we also compared the effects of raphin‐1 and ONC201 on their various downstream effectors in PED‐DHGG. As depicted in Fig. 6A,B, ONC201 decreased survival in both SU‐DIPG‐VI and KNS‐42. However, sensitivity to ONC201 was lower in KNS‐42 cells with mutated H3.3G34V than in SU‐DIPG‐VI cells with mutated H3.3K27M. Unlike raphin‐1, ONC201 did not decrease the level of CReP and did not alter the level of XBP1s in SU‐DIPG‐VI but led to a prominent decrease of XBP1s KNS42 (Fig. 6C,D). Nevertheless, in SU‐DIPG‐VI cells, both raphin‐1 and ONC201 treatments had similar effects on PeIF2α, GADD34, BiP, and DR5. Raphin‐1 consistently reduced ClpX levels by 35% ± 10 (*n* = 8; *P* < 0.001); however, its effect was modest compared to the more dramatic effect of ONC201. Importantly, salubrinal did not decrease the level of ClpX in any of the three cell lines included in our study (Figs 2E and 6C,D). Moreover, as shown in Fig. 2E, raphin‐1 decreased the level of ClpX in both MEF^WT^ and MEF^S51A^. These results suggest that the effect of raphin‐1 on the level of ClpX is independent of PeIF2α. In KNS‐42 cells, the effect of raphin‐1 and ONC201 on PeIF2α, GADD34, DR5, and CLpX was similar to that observed in SU‐DIPG‐VI cells, except that ONC201 did not affect the level of BiP (Fig. 6D).

Given that BiP and ClpXP play important roles in the maintenance of mitochondrial function and structure [21, 44], we also examined the effect of raphin‐1 on the mitochondrial ultrastructure in SU‐DIPG‐VI cells. As with a previously reported effect of ONC201 on mitochondria [43], raphin‐1 treatment also notably decreased the cristae and matrix contents in nearly all mitochondria (Fig. 6E).

### 3.6 Raphin‐1 increases the sensitivity of PED‐DHGG to radiation

**Fig. 7:**
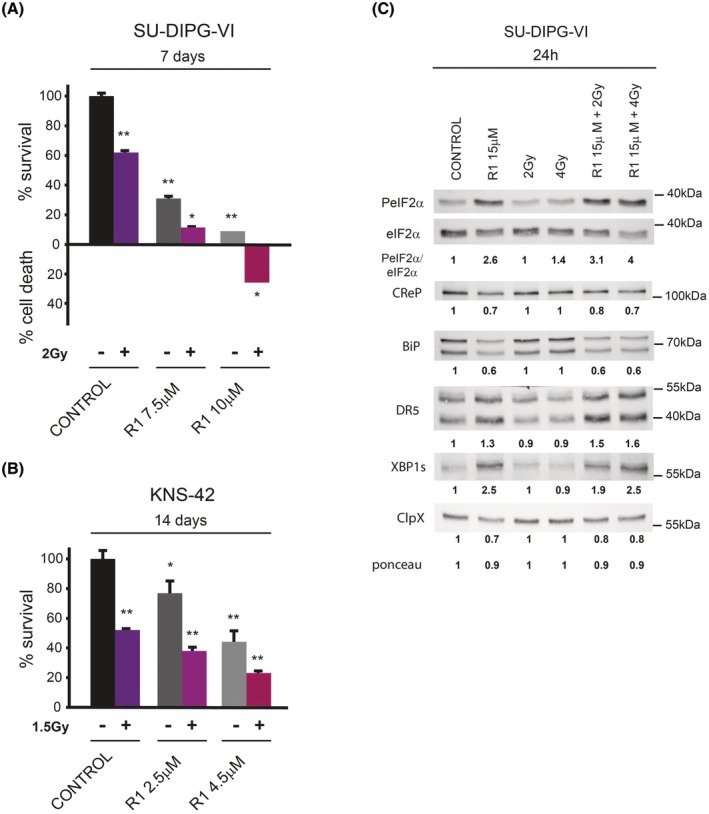
Raphin‐1 increases the sensitivity of PED‐DHGG to radiation. (A, B) SU‐DIPG‐VI and KNS‐42 cells were plated in triplicates and treated with raphin‐1 and radiation as described in Section 2. Values are mean survival (%) or cell death (%) ± SD of two independent experiments. The significance of the differences between treatments and control was determined using an unpaired Student *t*‐test – *P < 0.05, **P < 0.005. (C) SU‐DIPG‐VI cells were treated with 15 μm raphin‐1 and ionizing irradiation for 24 h and processed for western blot analysis as described in Section 2. Numbers at the bottom of the autoradiograms indicate treatment‐dependent changes in the level of the proteins and equal loading control (Ponceau). The experiment was reproduced once with similar results (*n* = 2). R1‐raphin‐1, Gy‐gray.

**Fig. 8:**
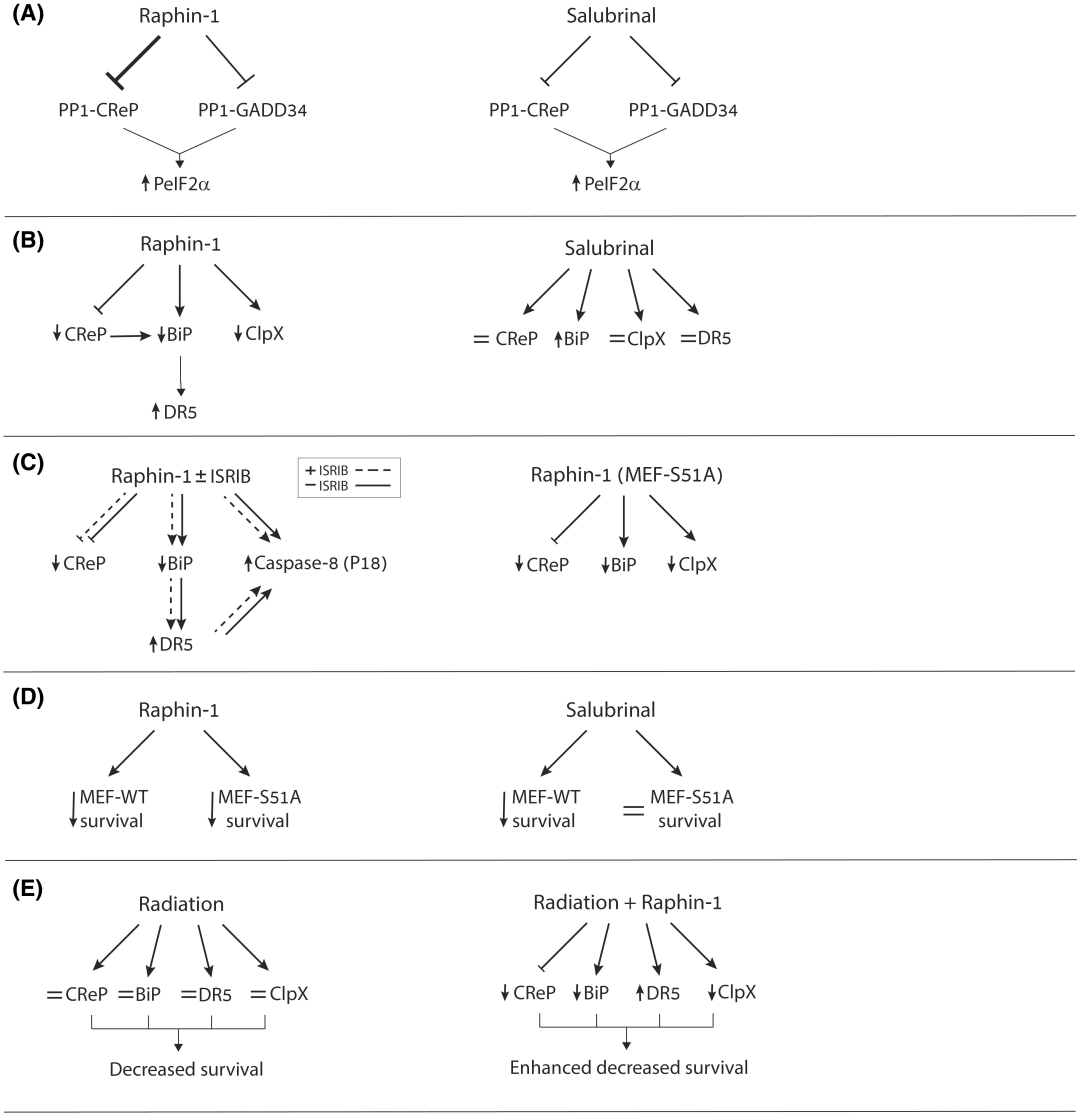
Raphin‐1 increases sensitivity of PED‐DHGG via PeIF2α‐independent mechanisms. (A) Both raphin‐1 and salubrinal inhibit PeIF2α phosphatase and increase the cellular level of PeIF2α. However, the affinity of raphin‐1 to CReP‐PP1 is higher than to GADD34‐PP1, as indicated by the thicker truncated line. (B) Despite the similar effect on increased PeIF2α, both drugs manifest different effect on shared molecular targets and different targets altogether. Raphin‐1 alters the conformation of CReP and reduces its level, a phenomenon that can lead to a decreased level of BiP. Reduced level of BiP leads to an increased level of DR5. (C) Experiments with ISRIB and MEF‐S51A show that the differential effect of raphin‐1 on downstream effectors is PeIF2α‐independent. (D) Unlike salubrinal, raphin‐1 can decrease PED‐DHGG survival by activating PeIF2α‐independent processes. (E) Raphin‐1 triggers PeIF2α‐independent processes that mediate its radiosensitizing effect on PED‐DHGG. = Indicates no change in protein level.

As we previously demonstrated [10] and as shown in Fig. 7A,B, and Fig. S4, raphin‐1 increased the sensitivity of PED‐DHGG to ionizing irradiation—presently the only standard of care available for this condition. Decreased levels of BiP and ClpXP activity or increased DR5 levels are all known to decrease cell survival and increase sensitivity to ionizing irradiation [45, 46, 47, 48]. Because these same factors were all affected by raphin‐1 treatment, we examined how raphin‐1 affects their expression in irradiated cells by co‐treating SU‐DIPG‐VI cells with raphin‐1 and clinically relevant radiation doses (e.g., 2 or 4 Gy). As depicted in Fig. 7C, radiation alone did not promote an increase in DR5 or XBP1s or a decrease in BiP or ClpX. By contrast, coadministration of radiation and raphin‐1 decreased CReP, BiP, and ClpX levels, increased DR5 and XBP1s levels, and enhanced eIF2α phosphorylation, suggesting that the activation of the unique molecular pathways modulated by raphin‐1 in irradiated SU‐DIPG‐VI cells was an underlying factor in its radiosensitizing effect. Fig. 8 illustrates our key findings, highlighting the involvement of PeIF2α‐independent mechanisms in mediating the effect of raphin‐1 on the sensitivity of PED‐DHGG to radiation.

## 4 Discussion

Several laboratories, including ours, have employed salubrinal and phosphomimetic [S51D] eIF2α to show that a sustained increase in PeIF2α decreases survival in cancer cells and increases their sensitivity to antineoplastic treatments [10, 49, 50, 51, 52, 53, 54]. We have also demonstrated that salubrinal inhibits DNA repair in breast cancer cells [51] and that, like salubrinal, raphin‐1 decreases PED‐DHGG survival, induces DNA damage, and enhances DNA damage in the presence of the PARP‐1 inhibitor niraparib [10, 52].

Recent studies have demonstrated that both GADD34 and CReP participate in cellular processes that are independent of their roles as regulators of PeIF2α phosphatase [19, 20]. These studies have localized GADD34 to the ER–Golgi intermediate domain and the Golgi, whereas CReP has been localized to the ER, cellular endosomes, and the plasma membrane [19, 20, 22]. In addition, although CReP is exclusively associated with cellular membranes, GADD34 is also found in the cytosol [55]. Therefore, our aim in this study was to determine whether the different interactions of raphin‐1 and salubrinal with CReP and GADD34, combined with the diverse functions of these proteins, would lead to different time‐dependent effects on shared molecular targets. We also hoped to uncover different molecular targets relevant to the survival of PED‐DHGG and their response to treatments.

While both drugs decreased CReP levels, the effect of salubrinal was short‐lived (dissipating by 48 h) and inversely related to the increased expression of GADD34, whereas the raphin‐1 effect was sustained throughout the 48‐h experiment. In addition, the two drugs exerted opposite effects on BiP protein levels. While raphin‐1 treatment promoted a time‐dependent decrease in BiP levels, salubrinal treatment caused a time‐dependent increase. The fact that raphin‐1 and salubrinal exerted different effects on CReP and BiP, despite inducing a similar increase in PeIF2α, is consistent with the observation that raphin‐1 maintained its effect on these two proteins in the presence of ISRIB. Importantly, experiments with MEF^WT^ and MEF^S51A^ showed that the differences between raphin‐1 and salubrinal effects on the cellular levels of CReP and BiP were independent of PeIF2α. Furthermore, in contrast to salubrinal, raphin‐1 could decrease cell survival in a PeIF2α‐independent manner. Our experiments indicated that the raphin‐1‐induced decrease in CReP involved increased CReP degradation. Conversely, the decreased level of BiP seemed to reflect its decreased translation, in agreement with the findings of Kastan et al. regarding the role of CReP in ER‐associated BiP translation [22].

Due to their elevated metabolic and proliferative rates, as well as exposure to unique environmental stresses, cancer cells exhibit a heightened reliance on BiP for the resolution of ER stress, surpassing that of most normal cells [56]. Beyond its function as a regulator of ER overload, BiP also facilitates survival pathways and inhibits apoptotic processes [57]. Recent studies have demonstrated that BiP is present in the nuclei of both lung cancer cells and stressed normal lung cells, where it modulates the transcription of the epidermal growth factor receptor by sequestering the transcriptional repressor inhibitor of DNA binding 2 [58]. Furthermore, BiP activity is essential for the expression of the *KRAS* ^*G12D*^ mutation in *KRAS* ^*G12D*^‐driven pancreatic and lung cancer cells [59]. BiP also plays a critical role in the regulation of mitochondrial metabolism. Specifically, BiP is localized to the mitochondria‐associated ER membrane subdomain, where it initiates the folding and mitochondrial transfer of steroidogenic acute regulatory protein, which is vital for mitochondrial steroidogenesis [60]. Through its interaction with the Sec61 complex, BiP prevents calcium ion (Ca^2+^) leakage into the cytosol following the insertion of newly synthesized polypeptides into the ER [61]. Additionally, by modulating Ca^2+^ efflux from the ER and its subsequent uptake by mitochondria, BiP reduces the production of reactive oxygen species in astrocytes' mitochondria under glucose and oxygen deprivation conditions [62]. BiP also protects cancer cells from apoptosis by forming inhibitory complexes with caspase‐7 [63]. As a result, increased BiP expression in various cancer cell lines and tumors is associated with enhanced cell survival and resistance to therapeutic interventions [21, 64]. Therefore, the differential effects of raphin‐1 and salubrinal on BiP expression are of significant relevance to both cell survival and resistance to treatments. Indeed, our experiments with HA15, a specific BiP inhibitor [35], demonstrate that BiP activity contributes to the survival of PED‐DHGG cells. To date, isolated natural products, as well as rationally designed synthetic compounds, are known to interact with and inhibit BiP or downregulate its transcription. These agents have been shown to decrease the survival of cancer cells, including glioblastoma (GBM), and increase their sensitivity to antineoplastic treatments both *in vitro* and in xenograft mice models *in vivo* [65]. However, to date, only one compound targeting the BiP pathway—IT‐139, developed for pancreatic cancer—has advanced to clinical trials [66].

The different time‐course effects of salubrinal and raphin‐1 on XBP1s expression in SU‐DIPG‐VI cells and the different effects of BiP on their capacity to increase XBP1s levels in KNS‐42 cells suggest that different molecular factors are involved in the boosting of XBP1s protein. Indeed, the interaction between BiP and IRE1α could be titrated down, not only by its decreased level and/or its increased association with unfolded/misfolded proteins, but also via its displacement from IRE1α by HSP47 [67]. In addition, IRE1α could be activated by direct interaction with the ribosome‐associated complex [68], and the outcome of its noncanonical RIDD activity is also affected by the regulation of the activity of RNA 2′,3′‐cyclic phosphate and 5′‐OH ligase [69], which of these mechanisms contributes to the differential effect of raphin‐1 and salubrinal on the expression of XBP1s remains to be determined.

The DR5 protein is associated with the activation of caspase‐8 and cell death [31, 32]. In PED‐DHGG, raphin‐1 increased the cellular levels of DR5 protein, whereas salubrinal did not, and the raphin‐1 effect on DR5 is associated with the activation of caspase‐8. Both the DR5 increase, and caspase‐8 activation were independent of ISRIB.

In addition to their ability to increase PeIF2α levels, raphin‐1 and ONC201 have similar effects on common downstream effectors. However, whereas ONC201 treatment dramatically decreased ClpX levels, raphin‐1 treatment induced only a relatively modest, but consistent, decrease in this protein in both PED‐DHGG and MEF^S51A^. Reflecting the reported effect of ONC201 on the integrity of mitochondria [43], raphin‐1 decreased mitochondrial cristae and matrix contents. ONC201 reduces the survival of cancer cells through multiple mechanisms, including the disruption of mitochondrial functions. However, the distinct sensitivity of H3K27M‐mutated PED‐DHGG to ONC201 primarily stems from the accumulation of 2‐hydroxyglutarate. This metabolite elevates the cellular levels of H3K27me3, which counteracts the unique gene expression profiles that support the survival of H3K27M‐mutated PED‐DHGG cells [41]. As previously noted, PED‐DHGG demonstrates resistance to radiation therapy [7, 8]. Both ONC201 and its derivative ONC206 decrease the survival of PED‐DHGG cell lines and inhibit tumor growth in xenograft models in mice, as well as in clinical trials involving pediatric patients [36, 37, 38]. These compounds effectively cross the blood–brain barrier and exhibit limited, manageable toxicity in both preclinical and clinical settings [41, 70]. The two drugs have been shown to increase the sensitivity of breast, pancreatic, and glioblastoma cell lines to radiation; however, a direct effect on the radiation sensitivity of PED‐DHGG cell lines has yet to be demonstrated. Interestingly, Zhou et al. reported that either ONC201 or ONC206 reduces the levels of O6‐methylguanine‐DNA methyltransferase in both glioblastoma and PED‐DHGG cells. They also observed decreased survival in glioblastoma cells when treated with a triple combination of temozolomide, ONC201 or ONC206, and radiation [71]. If these findings are validated in PED‐DHGG cells, they could expand the available treatment options for PED‐DHGG patients.

Recently, Werbrouck et al. reported that the presence of mutated TP53 in approximately 60% of patients further aggravated this innate resistance [72]. Our experiments showed that SU‐DIPG‐VI cells, which carry a gain‐of‐function mutation in TP53 [73], did not show decreases in BiP or ClpX in response to irradiation, nor did they show increases in the expression of XBP1s and DR5. Nevertheless, our results demonstrate that raphin‐1 can ‘impose’ these radiosensitizing changes on irradiated cells. Furthermore, decreased levels of BiP and ClpX and increased levels of DR5 are known to increase sensitivity to ionizing irradiation [45, 46, 47, 48], while forced activation of noncanonical RIDD during genotoxic stress has been reported to inhibit RIDD activities, which are required for DNA repair [74]. Increases in XBP1s levels in the face of reduced BiP in HEK‐293 cells have been demonstrated to inhibit autophagy—which may also increase sensitivity to radiation damage [75, 76]. DNA‐damaging agents, such as ionizing irradiation, doxorubicin, and etoposide, have been demonstrated to activate DR5 transcription in a wild‐type p53‐dependent manner [72]. However, certain drugs and cytokines, such as dexamethasone and interferon‐γ, activate DR5 transcription in cell lines that express mutated p53 [77]. Similarly, we demonstrated in this study that raphin‐1 increases DR5 levels in TP53‐mutated cells in a PeIF2α‐independent manner.

## 5 Conclusions

We have previously demonstrated that a sustained increase in PeIF2α decreases PED‐DHGG survival and increases their sensitivity to niraparib [10]. We have also shown that salubrinal and raphin‐1, which are both inhibitors of PeIF2α phosphatase, decreased PED‐DHGG survival and increased their sensitivity to ionizing irradiation. The present findings strongly indicate that raphin‐1 triggers radiosensitizing processes in PED‐DHGG in a PeIF2α‐independent manner.

Preclinical studies have demonstrated that raphin‐1 can cross the blood–brain barrier without causing adverse effects on body weight or long‐term memory in mice [16]. Further studies using mice with orthotopic PED‐DHGG tumors are needed to assess the potential effect of raphin‐1 on the radiation sensitivity of PED‐DHGG *in vivo*. Our findings indicate that raphin‐1 reduces the survival of PED‐DHGG cells harboring the H3K27M or H3G34R/V mutation through multiple mechanisms, some of which are known to have a greater impact on the survival of cancer cells compared to normal cells [56, 78]. This highlights the potential value of investigating the interactions of raphin‐1 and salubrinal with CReP and GADD34, as well as with other, yet unidentified, cellular components, to further the development of new therapeutic strategies for PED‐DHGG.

## Conflict of interest

The authors declare no conflict of interest.

## Author contributions

KE, SP, MY, and AT designed the study and analyzed the data; KE performed the experiments; ML analyzed the data; and SP, KE, and MY wrote the manuscript.

## Peer review

The peer review history for this article is available at https://www.webofscience.com/api/gateway/wos/peer‐review/10.1002/1878‐0261.70081.

## Supporting information


**Fig. S1.** BiP and XBP1s levels in PED‐DHGG cell lines. (A) KNS‐42 expresses a higher level of BiP than SU‐DIPG‐VI. (B, C) Early expression of XBP1s in SU‐DIPG‐VI and KNS‐42 in raphin‐1 and salubrinal‐treated cells. (A–C) SU‐DIPG‐VI and KNS‐42 cells were plated and treated for the indicated time with the specified concentrations of raphin‐1 or salubrinal and processed for western blot analysis as described in Section 2. Numbers at the bottom of the autoradiograms indicate treatment‐dependent changes in the level of BiP and XBP1s and the equal loading control (Ponceau). The experiments were reproduced once with similar results. R1‐raphin‐1, Sal‐ salubrinal.

**Fig. S2.** The effect of raphin‐1 and salubrinal on the level of CReP, Bip and GADD34 mRNA. SU‐DIPG‐VI cells were treated with raphin‐1 and salubrinal for the indicated time. RNA was extracted and treatment‐induced changes in mRNAs were evaluated by qRT‐PCR as described in Section 2. Data are mean relative quantification (RQ) ± SD of two independent experiments. Differences between treated and control cells were significant – *P < 0.05, **P < 0.005.

**Fig. S3.** S51D‐eIF2a, raphin‐1 and salubrinal decrease survival of PED‐DHGG. (A) KNS‐42 were plated in triplicates and transfected with HA‐tagged WT, S51A or S51D‐eIF2a [3] and counted 5 days later. Values are mean survival (%) ± SD of two independent experiments. (B) SU‐DIPG‐VI cells were plated in triplicates and treated with raphin‐1 and salubrinal as described in Section 2. Values are mean survival (%) ± SD of two independent experiments. Differences between treatments and control were significant – *P < 0.05.

**Fig. S4.** Raphin‐1 increases the sensitivity of KNS‐42 to radiation. Colony survival assay was conducted as described in Section 2. Numbers indicate the average colony numbers ± SD of two independent experiments.

## Data Availability

The data that support the findings of this study are available from the corresponding author [michal@droren.co.il] upon reasonable request.
