# Supplementary figures and images for: Raphin‐1 mediates the survival and sensitivity to radiation of pediatric‐type diffuse high‐grade glioma via phosphorylated eukaryotic initiation factor 2α‐dependent and ‐independent processes
Source: Mol Oncol. 2025 Jul 9;19(9):2648–69. doi: 10.1002/1878-0261.70081 (PMC12420360; doi:10.1002/1878-0261.70081)

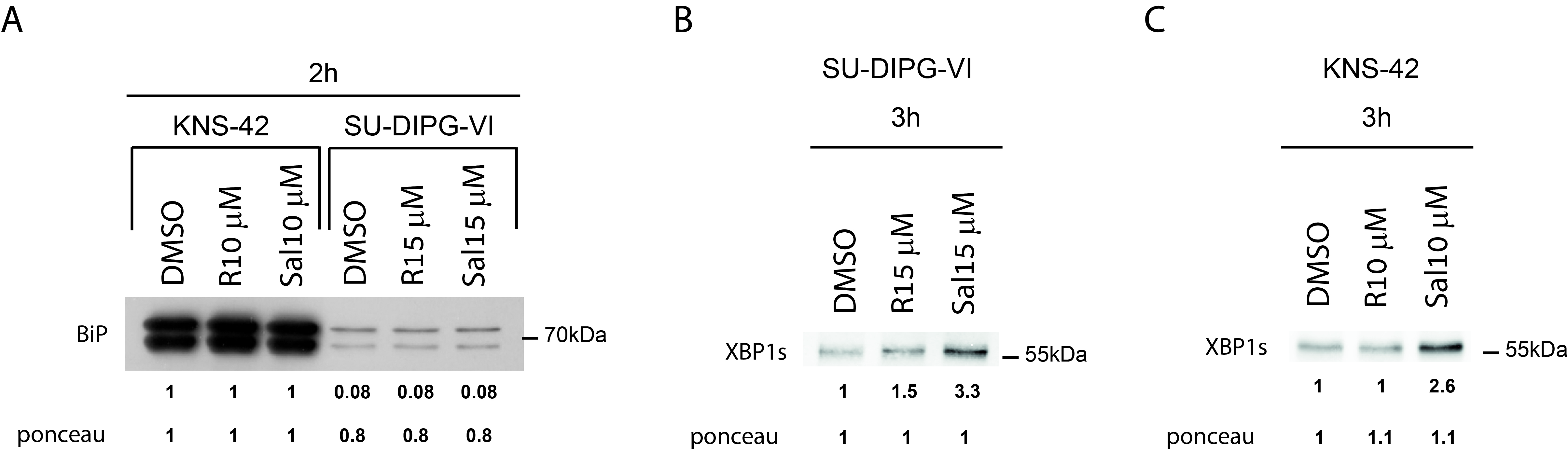

Supplement: Supplementary file 1 — Fig. S1. BiP and XBP1s levels in PED‐DHGG cell lines. (A) KNS‐42 expresses a higher level of BiP than SU‐DIPG‐VI. (B, C) Early expression of XBP1s in SU‐DIPG‐VI and KNS‐42 in raphin‐1 and salubrinal‐treated cells. (A–C) SU‐DIPG‐VI and KNS‐42 cells were plated and treated for the indicated time with the specified concentrations of raphin‐1 or salubrinal and processed for western blot analysis as described in Section 2. Numbers at the bottom of the autoradiograms indicate treatment‐dependent changes in the level of BiP and XBP1s and the equal loading control (Ponceau). The experiments were reproduced once with similar results. R1‐raphin‐1, Sal‐ salubrinal. [file MOL2-19-2648-s004.jpg]

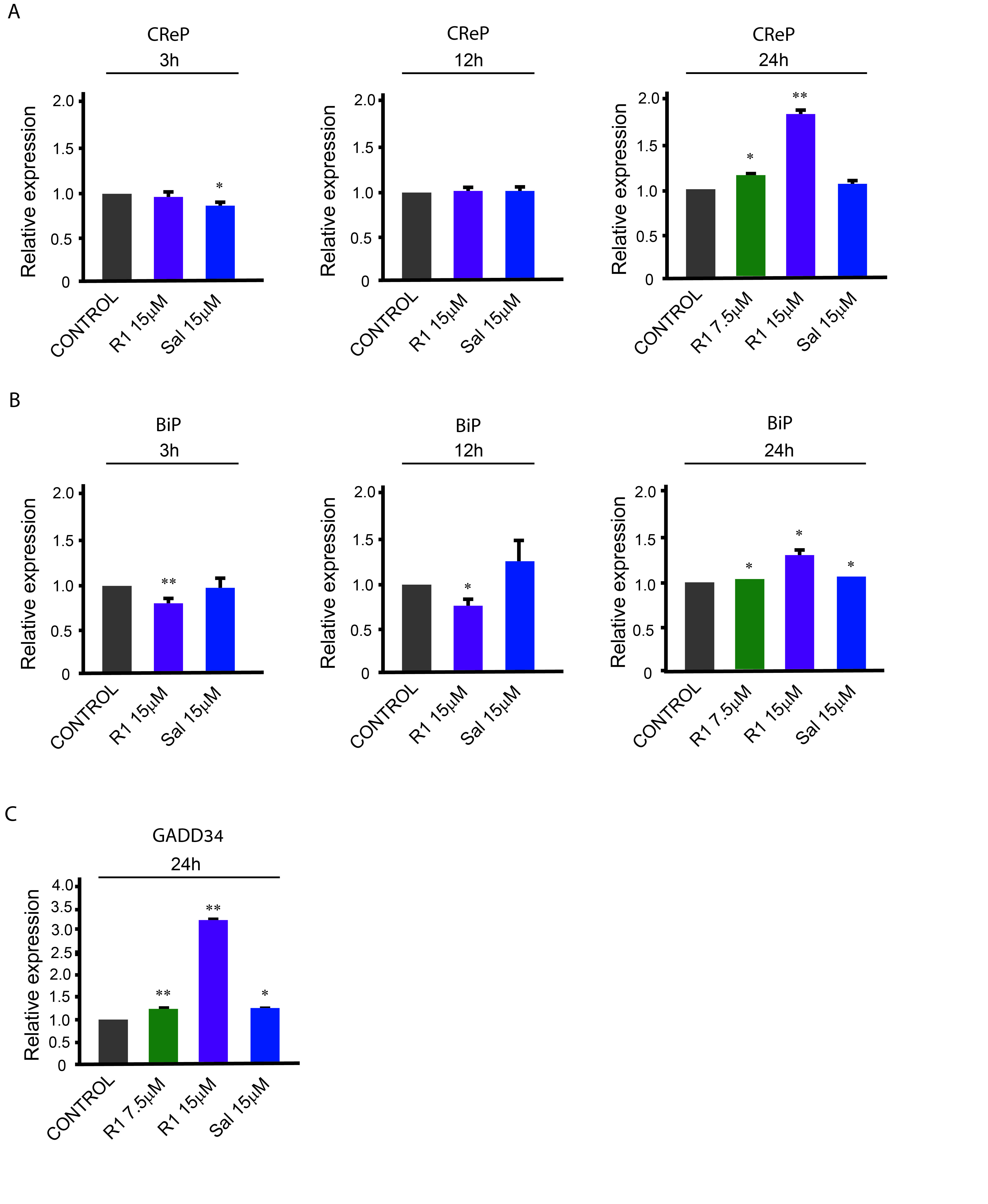

Supplement: Supplementary file 2 — Fig. S2. The effect of raphin‐1 and salubrinal on the level of CReP, Bip and GADD34 mRNA. SU‐DIPG‐VI cells were treated with raphin‐1 and salubrinal for the indicated time. RNA was extracted and treatment‐induced changes in mRNAs were evaluated by qRT‐PCR as described in Section 2. Data are mean relative quantification (RQ) ± SD of two independent experiments. Differences between treated and control cells were significant – *P < 0.05, **P < 0.005. [file MOL2-19-2648-s001.jpg]

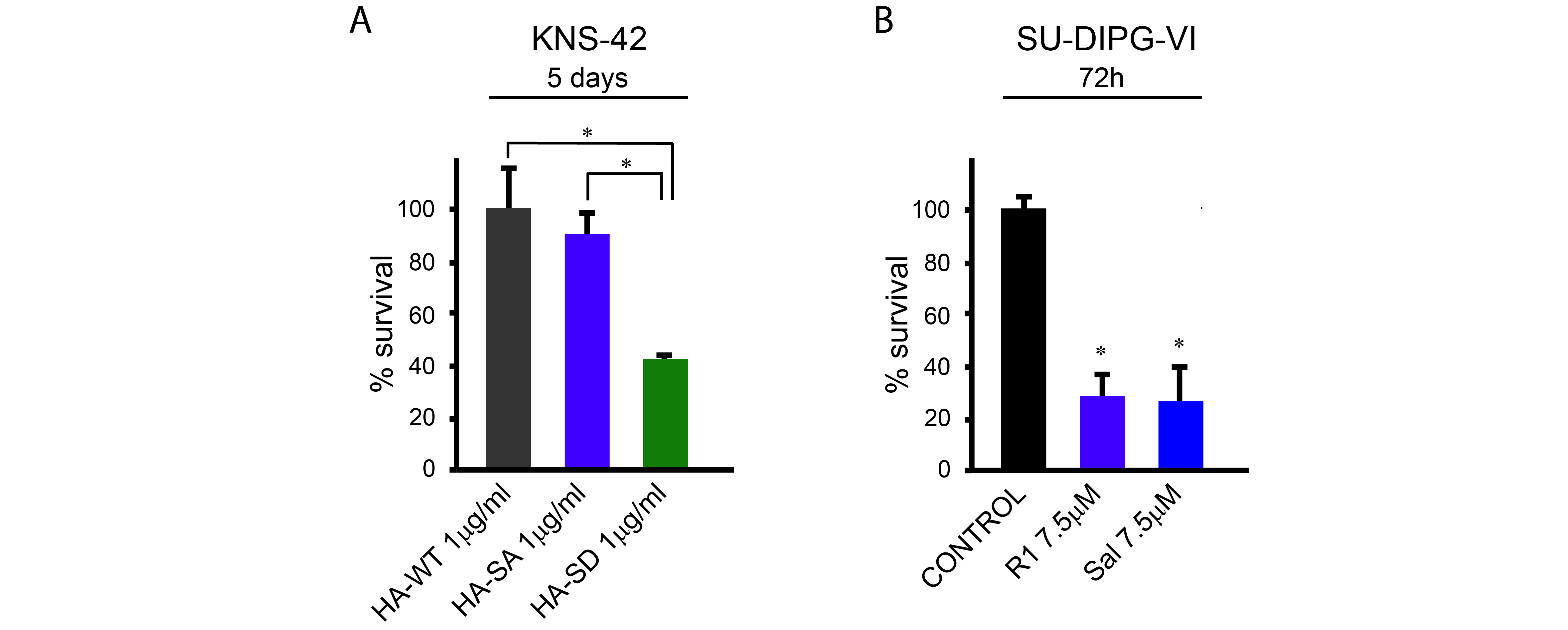

Supplement: Supplementary file 3 — Fig. S3. S51D‐eIF2a, raphin‐1 and salubrinal decrease survival of PED‐DHGG. (A) KNS‐42 were plated in triplicates and transfected with HA‐tagged WT, S51A or S51D‐eIF2a [3] and counted 5 days later. Values are mean survival (%) ± SD of two independent experiments. (B) SU‐DIPG‐VI cells were plated in triplicates and treated with raphin‐1 and salubrinal as described in Section 2. Values are mean survival (%) ± SD of two independent experiments. Differences between treatments and control were significant – *P < 0.05. [file MOL2-19-2648-s003.jpg]

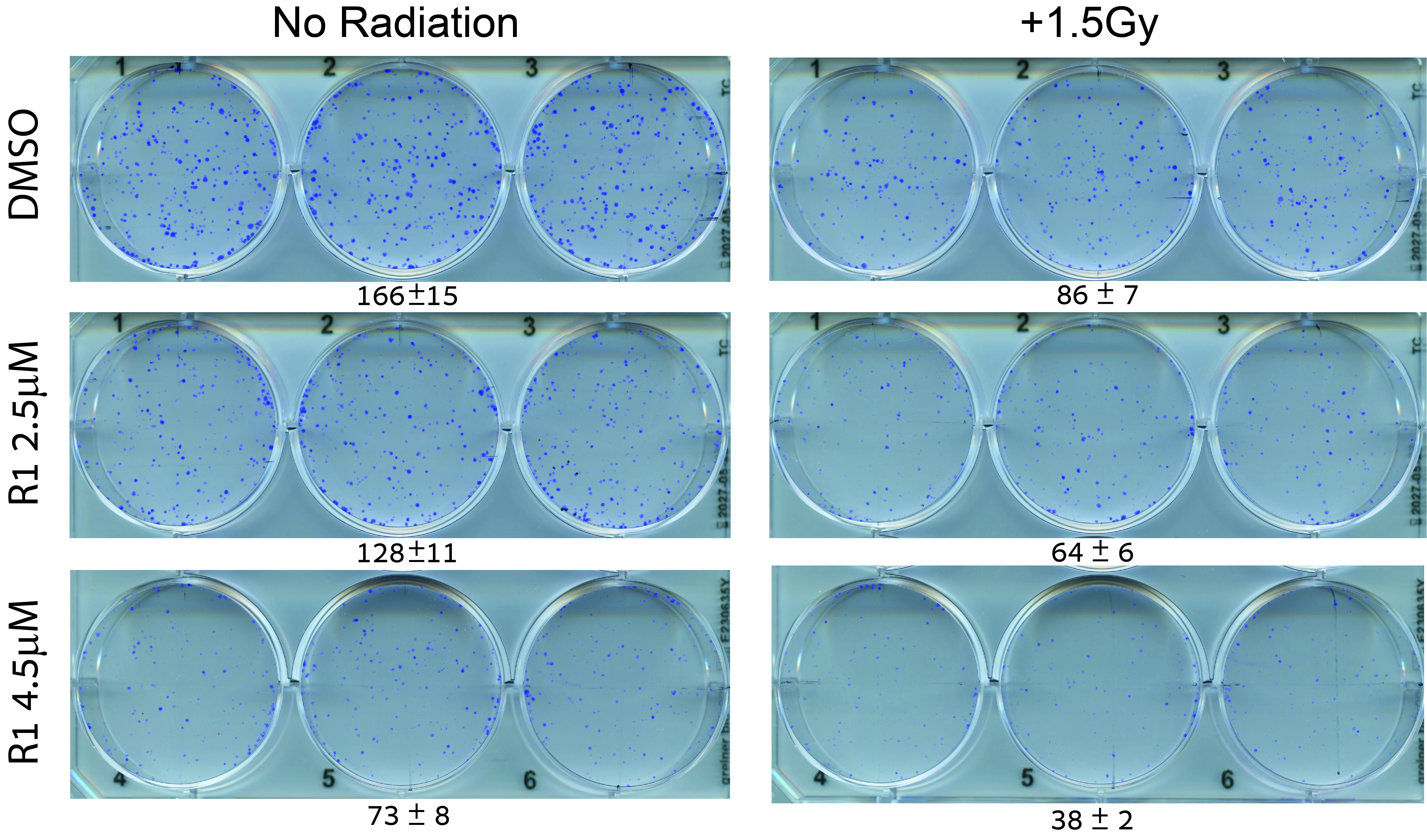

Supplement: Supplementary file 4 — Fig. S4. Raphin‐1 increases the sensitivity of KNS‐42 to radiation. Colony survival assay was conducted as described in Section 2. Numbers indicate the average colony numbers ± SD of two independent experiments. [file MOL2-19-2648-s002.jpg]
